# *G1* point mutation in growth differentiation factor 9 gene affects litter size in Sudanese desert sheep

**DOI:** 10.14202/vetworld.2021.104-112

**Published:** 2021-01-13

**Authors:** Amani Z. Abdelgadir, Lutfi M. A. Musa, Khaleel I. Jawasreh, Aubai O. Saleem, Faisal El-Hag, Mohamed-Khair A. Ahmed

**Affiliations:** 1 Department of Animal Production, Faculty of Agriculture, Omdurman Islamic University, Sudan; 2 Department of Animal Breeding and Genetics, Faculty of Animal Production, University of Khartoum, Sudan; 3 Arab Center for Studies of Arid Zones and Dry Land, The League of Arab states, Syria; 4 Department of Animal Productions, Faculty of Agriculture – Jordan University of Science and Technology, Jordan; 5 Department of Bioinformatics, Africa City of technology, Sudan; 6 Arid Land Research Center (ALRC), Tottori University, 1390 Hamasaka, Tottori 680-0001, Japan

**Keywords:** common ancestor, growth differentiation factor 9 gene, similarity and identity, Sudanese sheep

## Abstract

**Background and Aim:**

Sudanese desert sheep encompass different sheep breeds named according to the different Sudanese tribes that rear them such as the Dubasi, Shugor, and Watish sheep. The objectives of this study were to screen for *G1* point mutation in the polymorphic growth differentiation factor 9 (GDF9) gene, investigate its association with litter size, and construct the phylogeny of the different tribal breeds that belong to the Sudanese Desert sheep tribal types.

**Materials and Methods:**

Genomic DNA was extracted from whole blood of three tribal Desert sheep breeds (Dubasi, Watish, and Shugor) using the guanidine chloride method. Polymerase chain reaction-restriction fragment length polymorphism with *HhaI* restriction enzyme and sequencing techniques was used for genotyping the *GDF9* locus for possible mutations associated with litter size in the three desert sheep tribal types.

**Results:**

*G1* mutation in *GDF9* caused the replacement of Arginine by Histidine at residue 87. The wild type allele (A) had the highest frequency, whereas the mutant type allele (a) had the lowest in all the sequenced subtypes. The genotype frequencies of the wild type ewes (AA) were higher than the heterozygous (Aa) and the mutant type (aa) frequencies in the three studied desert sheep types. No significant differences were found in the allele frequency between the three tribal types. Litter size was significantly influenced by the genotypes of *GDF9* gene, parities, and subtypes (p≤0.01, 0.01, and 0.05, respectively). In the Watish sheep type, heterozygous sheep in their second parity recorded the highest litter size. Sequence alignment of *GDF9* gene samples with the database entry indicated that all three tribal types were similar and identical to the reference sequence. The phylogenetic tree revealed that Shugor is the common ancestor of the studied types and Watish is more closely related to Shugor than Dubasi. This result mi ght partly explain the lower reproductive performance of Dubasi compared to Watish and Shugor.

**Conclusion:**

The presence of one copy of *GDF9* gene increased litter size in the studied Sudanese Desert sheep. This locus may be used as a biomarker for litter size improvement through genotypic selection and allele or gene introgression.

## Introduction

Small ruminants, particularly native local breed types, play a significant socio-economic role in ensuring the livelihoods of a considerable portion of human populations in the tropics [1-3], and genetic manipulations in improving ewe production and reproduction present valuable opportunities to increase financial revenue [4-8]. Sudan harbors different sheep breeds referred to as the Sudanese Desert sheep which are distinguished according to the different Sudanese tribes that rear them such as the Dubasi, Shugor, and Watish breeds. These three sheep types are found in the central clay plain in Sudan under conditions of moderate rainfall (about 500 mm annually) and are mainly reared for meat production and significantly contribute to local consumption and export.

The growth differentiation factor 9 (*FecG, GDF9*) is a member of the transforming growth factor β released from oocytes during folliculogenesis. *GDF9* inhibits granulosa cell apoptosis and follicular atresia, both essential early for normal follicular development in sheep [9]. *GDF9* gene is also involved in cellular processes that regulate female sexual reproduction, gamete generation, gonad development, and ovulation cycle and modulates the signaling pathways of the transforming growth factor-beta receptor and the transmembrane receptor protein serine/threonine kinase [10].

Increasing meat production using scientific, accurate, and precise selective programs are one of the most important goals for genetic improvement in goats. These programs aim to identify and to correlate the genotypes of reproductive and productive traits of animals by determining polymorphisms and generating phylogenetic relationships [11-13]. The determination of gene polymorphisms is essential in farm animal breeding programs [11,14,15] to define genotypes of animals and their associations with productive, reproductive, and economic traits [16]. Species without sufficient genetic variability and diversity face more challenges in efficiently adapting to environmental fluctuations as they become less resilient and more susceptible to diseases [17,18]. Thus, preserving genetic diversity in indigenous breeds is a major concern during the design of selection programs to improve productive traits of a specific breed without diluting or losing other beneficial traits [7]. As conservation is dependent on deep and full knowledge of the genome of a specific breed [19], it is important to try to genetically characterize indigenous breeds [14] and to determine the applications of molecular genetics [20].

Hence, the objectives of the present study were to detect the *G1* point mutation of exon 1 of the *Ovis GDF9* gene and to investigate its association with litter size among the different tribal desert sheep breeds of Sudan (Dubasi, Shugor, and Watish).

## Materials and Methods

### Ethical approval

According to the Animal Use in Research Committee of the Sudan Veterinary Council, no special approval was required for this research. The guidelines of Sudan Veterinary Council regarding the handling of animals and sampling were strictly followed.

### Study period and location

The Genotypic Detection of *GDF9* Gene was carried out in the Molecular Biology and Immunology Unit, Department of Biology, Central laboratory of Veterinary Research, Ministry of Animal Resources and Fisheries, Sudan, from May 2017 to October 2019.

### Sample collection

Blood samples were randomly collected from 100 unrelated ewes of the three Sudanese Desert sheep tribal types [Dubasi (n=30), Shugor (n=30), and Watish (n=40)] from three states (Gezira, Sinnar, and Blue Nile). The blood samples were collected from the jugular vein in in 10 ml vacuum blood collection tubes containing EDTA. All samples were immediately placed on ice and transferred to the laboratory (The Central Laboratory of Veterinary Research, Sudan) where they were stored at −20°C until DNA extraction.

### DNA extraction

Genomic DNA was extracted from whole blood and the DNA purified using a modified version of the Guanidine Chloride method described by Zainabadi *et al*. [21]. Briefly, 3-5 ml blood was collected in EDTA tubes, and then 10 ml red cell lysis buffer was added in a Falcon tube and centrifuged for 5 min at 6000 rpm, this process was repeated until a clear pellet appeared. The supernatant was discarded, and 800 μL of white cell lysis buffer, 10 μL of proteinase K (10 mg/ml), 1 ml Guanidine Chloride, and 300 μL Ammonium Acetate were added, vortexed, and incubated at 37°C overnight. Then, an equal volume of chloroform in a new Falcon tube was added, mixed well and centrifuged at 6000 rpm for 5 min. The upper layer was transferred to a clean Falcon tube and 10 ml of cold ethanol (95%) was added and incubated at —20°C overnight. The sample was then centrifuged for 10-15 min at 6000 rpm and the supernatant was discarded. The pellet was washed with 4 ml of 70% ethanol and centrifuged for 7 min at 12000 rpm, and the supernatant discarded. The previous steps were repeated until the pellet became clear, at which point it was dried for 1-2 h, then dissolved in 100 ml of Tris EDTA buffer or 20 μL distilled water was added and stored at −20°C.

The quality of the extracted DNA was verified by adding 2 μL of template DNA and 3 μL of loading dye, and visualized on 1.5% Agarose gel with ethidium bromide. The images of each gel or the DNA band were photographed using Bio-Rad Gel Documentation 2000 system. Only samples of good quality were carried through for further analysis.

### Amplification and sequencing of the GDF9 gene

The amplification reaction was carried out by restriction fragment length polymorphism (RFLP)-Polymerase chain reaction (PCR) as described previously by Hanrahan *et al*. [22] and Jawasreh *et al*. [23] (Table-1) using 35 cycles at 95°C for 300 s for initial denaturation, followed by 94°C for 45 s for denaturation, 58°C for 40 s for annealing, 72°C for 60 s for extension, and a final extension at 72°C for 10 min. The amplified samples were loaded on 1.5% agarose gel and visualized under UV with the gel documentation system (Bio-Rad, USA).

**Table-1:** The primers and restriction enzyme used for the analysis of the *GDF9* gene at the *G1* point mutation using the HhaI restriction endonuclease.

Primer name	Primer sequences 5’-3’	Polymerase chain reaction product (bp)	Annealing temperature	Reference
G9-1734F	5’-GAAGACTGGTATGGGGAAATG-3’	462 bp	58º C	[4]
G9-2175R	5’-CCAATCTGCTCCTACACACCT-3’			[5]

PCR products (462 bp) were digested by *HhaI* restriction enzyme at 37°C for 3 h. 7 μL of PCP products were added to a solution of 2 μL buffer, 0.5 μL *HhaI* enzyme, and 0.5 μL bovine serum albumin (BSA) as enhancer, and topped with sterilized water to a final volume of 25 μL. The digestion reaction was then pulsed for 30 s, and incubated at 37°C overnight.

Further, the purification and standard sequencing of the PCR products of the *GDF9* gene was performed for the three genotypes of *GDF9* gene by Macrogen Company (Seoul, South Korea).

### Gene and genotype frequency estimation

Gene and genotype frequencies were calculated based on the counting method as described by Falconer and Mackay [24]. The Chi-square test was used to test the Hardy-Weinberg equilibrium.

### Association analysis between genotypes, parity number, and litter size in the three Desert sheep tribal types

The association between the detected genotypes of the studied fecundity gene (*GDF9**)***, parity number, and litter size in the three Desert sheep types was analyzed using the General linear model (GLM) method using IBM SPSS version 21 (IBM Corp., Armonk, N.Y., USA). The Chi-square test, analysis of variance (ANOVA), and Duncan multiple range test (DMRT) were used to check the effect of various factors on litter size and differences were considered significant at p<0.05.

The linear model (1):

Y_ijk_ = + B_i_ + G_j_ + P_k_ + E_ijk_

Where:

Y_ijk_= litter size record of the ijk^th^ ewe.

μ = overall mean.

B_i_= effect of the i^th^ sheep tribal type (i = 1-3).

G_j_= effect of the j^th^ genotype (j = 1-3).

P_k_= effect of the k^th^ parity number (k = 1-4).

E_ijk_= random error term.

### Bioinformatics analysis for sequence similarity and alignment

The DNA chromatograms were presented by Finch TV program version 1.4.0 (http://www.geospiza.com/Products/finchtv.shtml), and the DNA cleaning process was done by removing nucleotides with sharp quality (<10). The nucleic acid sequence of *GDF9* gene was blasted against nucleotide databases to get the most similar sequences [25]. Sequences with high identities were downloaded from NCBI in the FASTA format and aligned with the reference sequence (NC_019484) in Bio Edit 7.0 [26] from NCBI [27]. Global phylogenetic trees were generated using multiple sequence alignment by CLUSTALW [28] to determine the relationship among sheep breeds from the different tribes. The reference proteins of the studied fecundity genes were obtained from ExPASy-Universal protein resource [29]. Nucleotide sequence was translated to protein by ExPASy translation tool [29] and subjected to multiple sequence alignment using Bio Edit 7.0 software.

## Results

### Detection of the G1 mutation in the GDF9 gene

**Figure-1:**
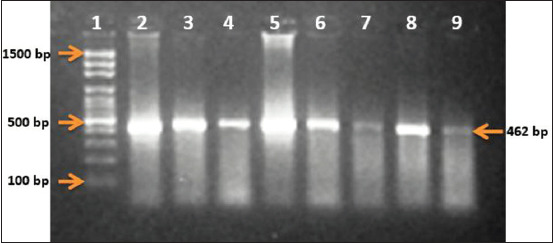
Polymerase chain reaction products of *GDF9* exon 1 (462 bp) on 1.5% agarose gel electrophoresis in Dubasi, Shugor, and Watish Sudanese Desert sheep tribal types. Lane 1, DNA ladder: MW 100-1500 bp fragments. Lane 2, 3, 4, 5, 6, 7, 8, and 9 showing typical band size of 462 bp corresponding to the molecular size of *GDF9* gene.

Amplification of the *GDF9* exon 1 showed the expected fragments with a product size of 462 bp fragment, as shown in Figure-1. Furthermore, the PCR-RFLP approach was applied to identify genotypes of the *GDF9* gene in the sheep samples using *HhaI* restriction enzyme. The nucleotide substitution G to A in *GDF9* exon 1 region disrupts the cleavage site of *HhaI* restriction enzyme (GCGC to GCAC) at nucleotide 260 of the 462 bp PCR product. The amino acid change was Arginine to Histidine at residue 87.

**Figure-2:**
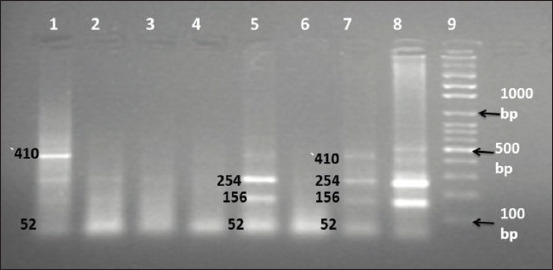
DNA electrophoretic pattern of *G1* point mutation of the *GDF9* gene amplified after digestion with *HhaI* restriction enzyme in the three Sudanese Desert sheep types. Lane 1, 7, and 8 (Aa): 410, 254, 156, and 52 bp. Lane 2, 3, 4, and 6 undigested PCR product. Lane 5 (AA): 254, 156, and 52 bp. Lane 9 DNA ladder: MW 100-1500 bp fragments.

**Figure-3:**
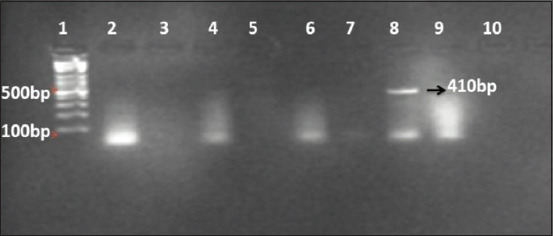
DNA electrophoretic pattern of *G1* point mutation of the GDF9 gene amplified after digestion with *HhaI* restriction enzyme. Lane 1, DNA ladder: MW 100-1500 bp fragments. Lane 2, 4, 6, and 9 undigested PCR product. Lane 3, 5, 7, and 10 empty. Lane 8 (aa): 410 and 52 bp.

The digestion of the PCR products by *Hha1* restriction enzyme showed three genotypes. Wild type (AA) had three fragments of 254, 156, and 52 bp, heterozygous (Aa) had four visible bands of 410, 254, 156, and 52 bp (Figure-2), and mutant types had two fragments of 410 and 52 bp (Figure-3).

### Genetic variability of the GDF9 gene

The allele and genotype frequencies of *GDF9* gene are shown in Table-2. The wild type allele (A) had the highest frequency, whereas the mutant type allele (a) had the lowest frequencies in Dubasi, Watish, and Shugor. The genotype frequencies of the wild type ewes (AA) were substantially higher than the heterozygous (Aa) and mutant type (aa) frequencies in the three populations of sheep tribal types.

**Table-2:** Allele and genotype frequencies of the point mutation *G1* of the *GDF9* gene in Dubasi, Shugor, and Watish sheep tribal types^1^.

Sheep tribal type	Number of animals	Allele frequency				Genotype frequency				H.W.E
			
	Total	AA	Aa	aa	(A)	(a)	AA	Aa	aa	(χ^2^- value)
Dubasi	26	16	8	2	0.77	0.23	0.61	0.31	0.08	0.63 NS
Shugor	28	16	10	2	0.75	0.25	0.57	0.36	0.07	0.16 NS
Watish	34	20	12	2	0.76	0.24	0.59	0.35	0.06	0.04 NS
Total	88	54	30	6	0.78	0.22	0.60	0.34	0.06	14.00 S

1 NS=No significant deviation from HWE, S=Significant deviation from HWE, (p<0.01)

Chi-square test was used to assess the Hardy-Weinberg equilibrium. Chi-square test showed that the analyzed populations of Dubasi (χ^2^=0.63), Shugor (χ^2^=0.16), and Watish (χ^2^=0.04) were in the Hardy-Weinberg equilibrium, while the pooled population of the three desert sheep tribal types was not (χ^2^=14.00).

### Association of the GDF9 gene with litter size

The least squares means and standard errors for litter size of the different *GDF9* genotypes and parities in Dubasi, Shugor, and Watish desert sheep are presented in Table-3. The ewes with heterozygous (Aa) and homozygous wild type (AA) genotypes had 0.346 and 0.207 more lambs than the homozygous (aa) genotypes, respectively. Moreover, the Watish desert sheep had the highest litter size, followed by Shugor and Dubasi. The second parity was higher in litter size followed a lower parity sequentially in the 3^rd^, 4^th^, and 1^st^ parities.

**Table-3:** Association between *GDF9* genotypes and parity number with litter size trait in Dubasi, Shugor, and Watish desert sheep tribal types^1^.

Variable	Type/Number	Litter size
Sheep tribal type	Dubasi	1.134±0.053^a^
	Shugor	1.232±0.053^ab^
	Watish	1.307±0.051^b^
Genotype	AA	1.247±0.033^b^
	Aa	1.386±0.046^b^
	Aa	1.040±0.094^a^
Parity number	1^st^	1.130±0.053^a^
	2^nd^	1.357±0.053^b^
	3^rd^	1.211±0.058^a^
	4^th^	1.201±0.072^a^
Grand mean		1.224±0.037

1 Means with same superscripts within each item are not significantly (p<0.05) different

ANOVA analysis of litter size in the three desert sheep tribes is presented in Table-4. Litter size was highly significantly influenced by the genotypes of *GDF9* gene, parities, and sheep tribe (p<0.01).

**Table-4:** Analysis of variance of the effect of breed, *GDF9* genotypes, and parity on litter size in the three desert sheep tribes^1^.

Source of variation	DF	SM	MS	F-ratio	Pr>F
Sheep tribe	2	1.472	0.736	3.957*	0.020
Parity	3	2.364	0.788	4.234**	0.006
Genotypes	2	2.411	1.205	6.479**	0.002

1 SM=Sum of squares, DF=Degrees of freedom, M=Mean of squares.

* p<0.05,

** p<0.01

### DNA sequencing of the GDF9 gene

**Figure-4:**
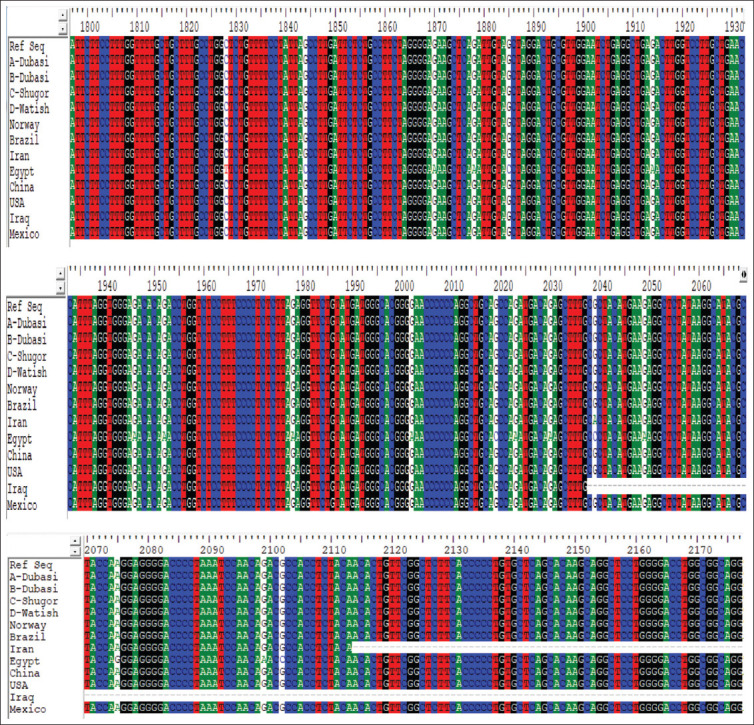
Similarity and identity of alignments between the *GDF9* DNA sampled from the three Desert sheep tribes and the database Ref Seq (Bio-Edit 7.0 software), Iraq (MF416087), Norway (HE866499), Egypt (KT357485), Brazil (FJ429111), Iran (KX377509), China (KR063137), USA (AF078545), and Mexico (KT8530).

Four different samples of DNA were not used in the restriction enzyme analysis, but rather were subjected to sequencing. The objective was to broaden the sample base by including A-Dubasi (MN862511); B-Dubasi (MN862512); C-Shugor (MN862513); and D-Watish (MN862514). The results of the alignment of *GDF9* samples with the Ref Seq nucleotide database using Bio-Edit 7.0 software [26] indicated that all three desert sheep tribal types (Dubasi, Shugor, and Watish) were similar and identical to the reference sequence. No nucleotide changes or mutations were detected in these four samples (Figure-4).

**Figure-5:**
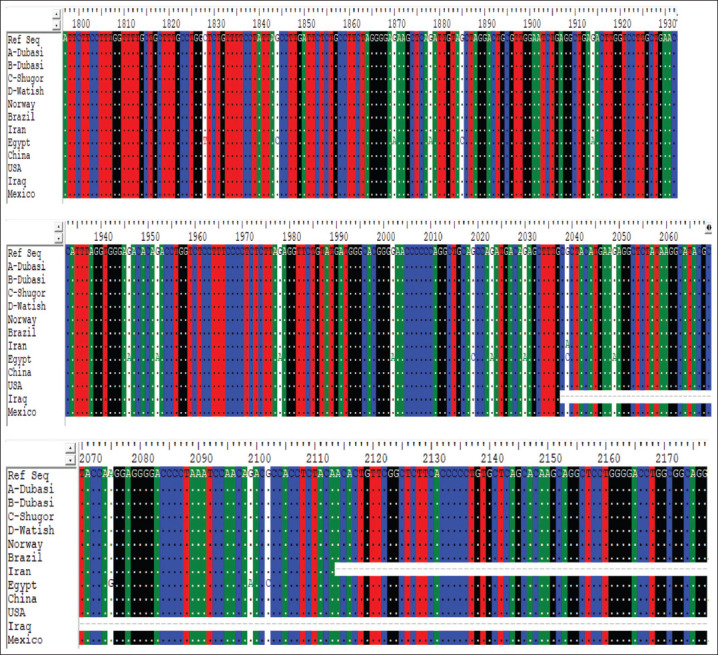
Conserved regions in the alignment of *GDF9* gene with Ref Sequence (Bio-Edit 7.0 software), Iraq (MF416087), Norway (HE866499), Egypt (KT357485), Brazil (FJ429111), Iran (KX377509), China (KR063137), USA (AF078545), and Mexico (KT8530).

All Sudanese breeds showed conserved regions with the reference sequence (Figure-5).

**Figure-6:**
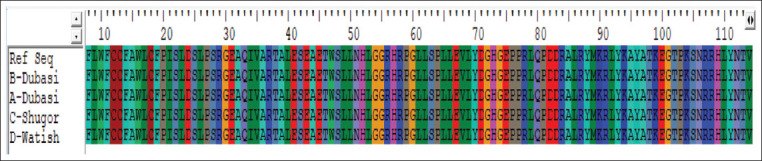
Amino acid alignment of *GDF9* gene with ref sequence using Bio-Edit 7.0 software.

No changes were detected as all translated amino acids were similar to the reference sequence without any mutation, as shown in Figure-6.

**Figure-7:**
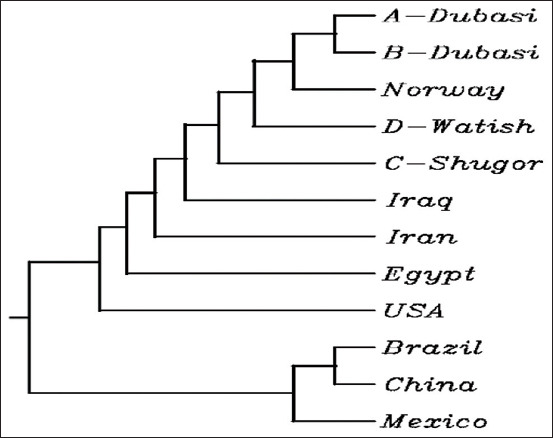
Global Phylogenetic tree (Multiple sequence alignment by CLUSTALW). Iraq (MF416087), Norway (HE866499), Egypt (KT357485), Brazil (FJ429111), Iran (KX377509), China (KR063137), USA (AF078545), and Mexico (KT8530).

Figure-7 shows the phylogenetic tree of the three breeds. The two samples of Dubasi tribal type (A and B) were the most closely related. Norway was the closest global sample to Sudanese Desert sheep (Including Watish and Shugor). Brazil, China, and Mexico were outgroup as the least related, where China and Brazil were more related to each other than the Mexican samples.

## Discussion

**Figure-8:**
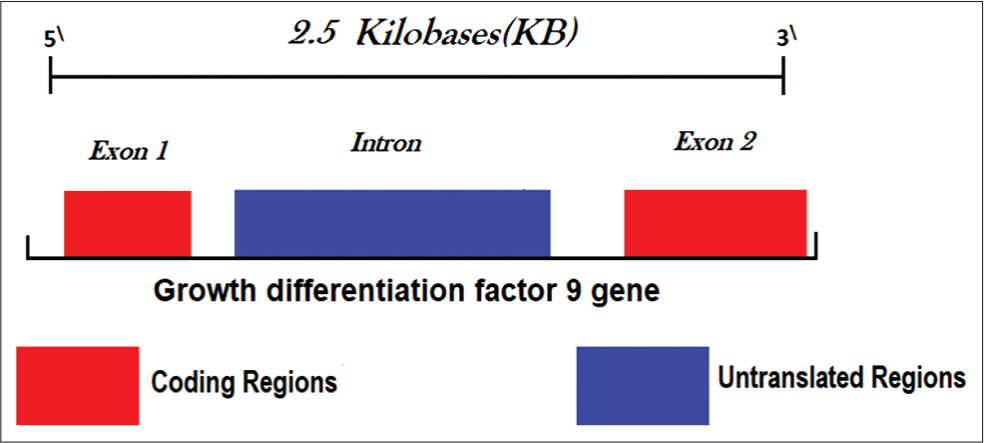
*GDF9* gene structure with two exons separated by one intron [33].

**Figure-9:**
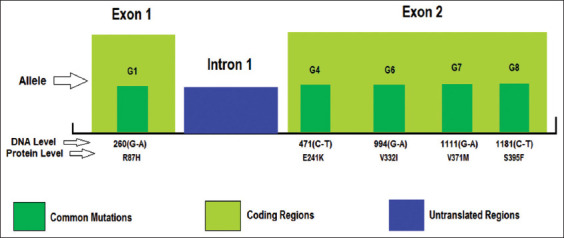
The most common mutations of *GDF9* gene. G2 and G3 located in the intron, while G5 in the exon without any changes in amino acid sequence [22].

The Ovine *GDF9* gene spans approximately 2.5 kb on chromosome 5 and contains two exons separated by one intron (Figure-8). The two exons spanned approximately 397 and 965 bp, respectively. Exon 1 encodes for 1-134 amino acids and exon 2 for 135-456 amino acids, while the intron spans about 1126 bp [9,30]. Mutations in *GDF9* have different effects on ovulation rate and can even cause infertility in some cases [31]. There are eight mutations (*G1*-*G8*) in the *GDF9* gene, where two of these mutations (*G2* and *G3)* are located in the intron, while *G5* located in exon 2, but do not result in amino acid changes [32] (Figure-9). The remaining five nucleotide substitutions (*G1, G4, G6, G7*, and *G8*) lead to amino acid substitutions. The *G1* mutation in heterozygote ewes showed the highest fertility, whereas the mutant types had a non-additive effect on ovulation rate, ultimately causing sterility [22]. The effect of this mutation (*FecG1*) is about 1.4 lambs per ewes. Barzegari *et al*. [33] attributed the absence of this gene to impaired follicular growth at the primary stage in homozygotes, thus resulting in sterility, while the inactivation of only one copy of *GDF9* increased the ovulation rate. *GDF9* gene could also influence the ovulation rate in a dose-dependent manner [34].

In the current study, we analyzed the polymorphic variations of the gene coding for the *GDF9*, a member of the transforming growth factor β superfamily. This superfamily has a vital role in female fertility and the *GDF9* protein is essential for ovarian follicular development in sheep, especially during the early stages of folliculogenesis [35]. This study focused on scanning for the *G1* point mutation located in exon 1 of the *GDF9* gene in three Sudanese Desert sheep tribes.

PCR-RFLP, a simple and dependable method, was previously used to study the polymorphisms of the *GDF9* gene in many sheep breeds. In this study, the *G1* mutation in Dubasi, Shugor, and Watish Desert sheep tribal types was scanned and results showed that the frequency of the mutant allele was low (0.22) compared to the wild type allele (0.78) in the three studied populations. However, frequencies might change for a larger sample number since the *GDF9* locus is polymorphic. This observation is in agreement with frequencies observed in Belclare and Cambridge breeds [22], Arkha Merino sheep [36], Moghani and Ghezed breeds [33], Sangsari sheep [37], Baluchi sheep [23], Hisar sheep [38], Moghani sheep [39], and Saidi sheep [40]. On the other hand, Gorlov *et al*. [41] reported that the frequency of the mutant allele was high and no wild-type genotypes (AA) were detected in Volgograd and Salsk sheep, as was the case in Lori sheep [42,43]. Whereas Elfiky *et al*. [40] reported that all of the observed genotypes were wild type in Ossimi sheep. The low frequency of the mutant genotype found in the studied Sudanese sheep suggests the possibility of mitigating this important mutation in these populations using marker-assisted selection.

The Chi-square analysis indicated that the populations of Dubasi, Shugor, and Watish sheep were in Hardy-Weinberg equilibrium for the *GDF9* locus when considered separately, while they were not in equilibrium when pooled together. This result is probably due to the low sample size used in this study. Similar to this report, Nanekarani *et al*. [43], Bahrami *et al*. [44], and Kasiriyan *et al*. [37] reported that populations of Lori, Hisari, and Sangsari sheep were in Hardy-Weinberg equilibrium at the *GDF9* locus.

Several reports [45-47] on the inheritance of sheep litter size suggest that litter size varies between and within sheep breeds and it is regulated by ovulation rate and the number of inseminated oocytes [48]. In this study, litter size was significantly affected by *G1* point mutation of *GDF9* gene, where heterozygous genotypes presented higher litter size (1.386±0.046) compared to the wild-type genotype (1.247±0.033) and homozygous carrier ewes (1.040±0.094). This result is in agreement with those obtained in Belclare and Cambridge breeds [22], Romanov sheep [23], and Mongolia sheep [49]. However , Liandris *et al*. [42] reported a non-significant association between the mutation in the *GDF9* gene and litter size in Karagouniki breed.

The study showed that the Watish desert sheep type demonstrated the highest litter size (1.307±0.051) compared to Shugor (1.232±0.053) and Dubasi (1.134±0.053). The high occurrence of this mutation in Watish may explain the multiple births usually observed in this breed. Although, the low averages of litter size may also be a reflection of poor management standards in the study area.

In the present study, the average litter size in the second parity (1.357±0.053) was significantly higher than the other ones. The mean litter sizes of ewes in the third parity (1.211±0.058) and in the fourth parity (1.201±0.072) were not statistically different, but were higher than those in the first parity (1.130±0.053) (p<0.01). This result might be attributed to the degree of development of ewes in the first parity or to nutrition levels in the breeding season of the selected ewes. The amount of available pasture depends directly on the intensity of the rainy season which varies from 1 year to the other.

In the sequence analysis, a different set of four samples was used as mentioned previously for the purpose of expanding and generalizing the results. Our findings showed that there were no amino acid changes such as addition, substitution, or deletion in the tested samples of Sudanese breeds and therefore, the protein function was properly maintained. Moreover, alignment results of *GDF9* gene samples with the database sequence indicated that all the Sudanese types (Dubasi, Shugor, and Watish) were similar to the reference sequence. On the other hand, the phylogenetic tree analysis (Figure-9) showed that the USA sample (AF078545) is the most recent common ancestor of the Egypt (KT357485), Iran (KX377509), Iraq (MF416087), Shugor (C), Watish (D), Norway (HE866499), and Dubasi subgroup (A and B) samples, whereas Brazil (FJ429111), China (KR063137), and Mexico (KT8530) were out grouped. According to this finding, Shugor is the common ancestor of the three studied Sudanese Desert sheep breeds, and Watish is more closely related to Shugor than Dubasi. This result may explain the lower reproductive potential of the Dubasi compared to Watish and Shugor tribal Desert sheep breeds.

## Conclusion

The results of the present study revealed that the presence of one copy of *GDF9* gene increased litter size in the studied Sudanese Desert sheep and may be used for future improvement of litter size by means of genotypic selection and allele or gene introgression.
